# Meta-analysis of diagnostic accuracy of nucleic acid amplification tests for abdominal tuberculosis: A protocol

**DOI:** 10.1371/journal.pone.0243765

**Published:** 2020-12-14

**Authors:** Yanqin Shen, Likui Fang, Bo Ye, Guocan Yu

**Affiliations:** Zhejiang Tuberculosis Diagnosis and Treatment Center, Zhejiang Chinese Medicine and Western Medicine Integrated Hospital, Hangzhou, Zhejiang, China; International Medical University, MALAYSIA

## Abstract

**Background:**

Abdominal tuberculosis is a severe extrapulmonary tuberculosis, which can lead to serious complications. Early diagnosis and treatment are very important for the prognosis and the diagnosis of abdominal tuberculosis is still difficult. This study aims to evaluate the diagnostic accuracy of nucleic acid amplification tests (NAATs) for abdominal tuberculosis using meta-analysis method.

**Methods:**

We will search PubMed, the Cochrane Library, Embase, China National Knowledge Infrastructure, and the Wanfang database for studies evaluating the diagnostic accuracy of NAATs for abdominal tuberculosis until May 2020. We will include a systematic review and meta-analysis that evaluated the accuracy of NAATs for abdominal tuberculosis. Any types of study design with full text will be sought and included. The risk of bias will be assessed using the Quality Assessment of Diagnostic Accuracy Studies tool. Stata version 15.0 with the midas command packages will be used to carry out meta-analyses.

**Results:**

The results will provide clinical evidence for diagnostic accuracy of NAATs for abdominal tuberculosis, and this systematic review and meta-analysis will be submitted to a peer-reviewed journal for publication.

**Conclusion:**

This overview will provide evidence of NAATs for diagnosis of abdominal tuberculosis.

**Systematic review registration:**

INPLASY202060030.

## 1. Introduction

Tuberculosis (TB) is a serious threat to global health [1]. Severe types of extrapulmonary tuberculosis (EPTB) increase tuberculosis-related mortality, especially in immunodeficient populations. Abdominal tuberculosis is a common serious EPTB [2]. Abdominal tuberculosis can cause many complications, such as intestinal obstruction, intestinal perforation, which seriously affect the quality of life and prognosis of patients [3]. Therefore, early diagnosis and treatment of abdominal tuberculosis is very important to reduce the incidence of serious abdominal complications. Crohn’s disease (CD), inflammatory bowel disease (IBD) and abdominal tuberculosis have similar clinical presentations and pathologies [4]. It is easy to misdiagnose abdominal tuberculosis as CD and IBD, thus delaying the treatment. The diagnosis of abdominal tuberculosis is still challenging.

Nucleic acid amplification tests (NAATs) play a huge role in the diagnosis of microbiological infections, making it faster and more accurate [5]. NAATs are widely used in the diagnosis of tuberculosis, which make the early diagnosis of tuberculosis possible [6, 7]. In the diagnosis of EPTB, NAATs are also fast, accurate and efficient, and they improve the detection rate of tuberculosis, especially in specimens with low bacterial content, such as lymph nodes tuberculosis and tuberculous meningitis [8, 9]. Abdominal tuberculosis is a type of paucibacillary EPTB and NAATs also have these advantages in its diagnosis. However, the diagnostic efficacy of NAATs for abdominal tuberculosis remains controversial. The aim of this systematic review and meta-analysis is to assess the diagnostic validity of NAATs for the diagnosis of abdominal tuberculosis.

## 2. Methods

### 2.1 Design and registration

We will conduct a systematic review and meta-analysis of diagnostic test accuracy to assess the diagnostic effectiveness of NAATs for abdominal tuberculosis. We have registered the protocol on the International Platform of Registered Systematic Review and Meta-analysis Protocols (INPLASY), and the registration number is INPLASY202060030 [10]. The Preferred Reporting Items for Systematic Reviews and Meta-Analysis for Diagnostic Test Accuracy (PRISMA-DTA) guideline will be followed for reporting our systematic review [11].

### 2.2. Information sources

PubMed, the Cochrane Library, Embase, China National Knowledge Infrastructure (CNKI), and the Wanfang database will be searched for studies that evaluate NAAT’s diagnostic accuracy for abdominal tuberculosis until May 2020.

### 2.3. Search strategy

The search strategies will be conducted by Yanqin Shen and Likui Fang.

There will be no language restriction on our search. Search strategy of PubMed will be as follows:

#1 "Tuberculosis, Gastrointestinal"[Mesh] OR "Gastrointestinal Tuberculosis" OR “Intestinal tuberculosis" OR "Peritonitis, Tuberculous"[Mesh] OR "Tuberculosis, Peritoneal" OR "peritoneal tuberculosis" OR "Tuberculous ascites" OR "Tuberculous Peritonitis" OR "abdominal tuberculosis" OR "intra-abdominal tuberculosis"
#2 ("Nucleic Acid Amplification Techniques"[Mesh] OR "Polymerase Chain Reaction"[Mesh] OR "Real-Time Polymerase Chain Reaction"[Mesh] OR "Reverse Transcriptase Polymerase Chain Reaction"[Mesh] OR "Multiplex Polymerase Chain Reaction"[Mesh] OR "genexpert"[tw] OR Xpert OR "genotype"[tw])
#3 #1 AND #2

Similar search formulae will be used for Embase, the Cochrane Library, CNKI, and Wanfang databases.

### 2.4. Eligibility criteria

#### 2.4.1. Type of study

Any types of studies can be included, such as retrospective studies, prospective studies, case-control studies. We will include original researches with full text that assessed the diagnostic accuracy of NAATs for abdominal tuberculosis. The reference standard should be appropriate and precisely defined in the study. True positive (TP), false positive (FP), false negative (FN), and true negative (TN) values are provided directly in the articles or contain necessary data to calculate these values. We will exclude articles reported in languages other than Chinese and English, case reports, studies with a specimen size of less than 10, conference coverages, and studies with abstracts but no full text.

#### 2.4.2. Patients

We will include studies, which contain patients diagnosed with abdominal tuberculosis through NAATs. We will have no restrictions on age, gender, and nations.

#### 2.4.3. Index tests

NAATs will be considered as index test.

#### 2.4.4. Reference standards

Bacteriological confirmation of Mycobacterium tuberculosis (positive culture of Mycobacterium tuberculosis and/or microscopic identification of acid-fast bacilli on stained specimen smear) is reference gold standard.

*Composite Reference Standard (CRS)*. Radiological characteristics (such as tree-in-bud pattern and cavity) and histopathological features of the suspected tissue specimen (features of chronic granulomatous inflammation with caseous necrosis/ caseating granuloma). Positive of reference standard test and/or positive of all CRSs mentioned will be considered abdominal tuberculosis. If all factors are negative, it will be considered as non- abdominal tuberculosis.

#### 2.4.5. Literature screening and selection

Primary search results matching the search strategy will be imported into the ENDNOTE X9.2 literature management software. Two investigators (Yanqin Shen and Likui Fang) will screen candidate studies independently by reviewing the titles and abstracts followed by the full text. Disagreements between two the researchers will be resolved by discussion with a third researcher (Guocan Yu).

#### 2.4.6. Data extraction

Name of first author; year of publication; country of study; reference standard; TP, FP, FN, and TN values of the test; method of patient selection; test method; subtypes of abdominal tuberculosis (such as omental and visceral tuberculosis), type of specimen; specimen processing procedures (e.g., homogenization) and specimen condition along with other parameters will be extracted. The same two researchers will independently extract relevant data from each included study and cross-check their respective information. Disagreements between two the researchers will be resolved by discussion with a third researcher, similar to that used during the literature selection phase. In the same study, data against different reference standards will be treated separately.

#### 2.4.7. Quality evaluation

The two researchers will assess the quality of the relevant literature using a revised tool for Quality Assessment of Diagnostic Accuracy Studies (QUADAS-2) [12] separately for the different reference standards and the disagreements between researchers will be solved by discussion with a third researchers (Guocan Yu).

According to the PRISMA-DTA statement, systematic review and meta-analysis of diagnostic test accuracy studies was not required to assess publication bias.

#### 2.4.8. Data synthesis and statistical analysis

TP, FP, FN, and TN values will be obtained from each included study, and the estimated pooled sensitivity and specificity of NAAT for abdominal tuberculosis associated with the 95% confidence interval (CI) will be calculated against culture or CRS, using bivariate random-effects models. We will generate forest plots for sensitivity and specificity for each study and calculate the areas under summary receiver operating characteristic (SROC) curves (AUC). We will assess heterogeneity between studies using *I*^2^ statistics. An *I*^2^ value of 0% will be indicative of no heterogeneity, while a value greater than 50% will indicate significant heterogeneity [13]. If the necessary data are available, subgroup analyses will be done to evaluate the diagnostic accuracy of NAATs for abdominal tuberculosis, such as different test methods, subtypes of abdominal tuberculosis, types of specimen, methods of patient selection (consecutive or convenience), methods of decontamination (with or without N-acetyl-L-cysteine/sodium hydroxide), conditions of sample (frozen or fresh), homogenization methods (mechanical or otherwise). If the heterogeneity is obvious, meta-regression analyses and sensitivity analysis will be used to explore the source of heterogeneity. The meta-analysis for predefined variable types will be performed using at least four published studies. We will analyze the data from studies against CRS and culture separately. We will use Stata version 15.0 (Stata Corp., College Station, TX, USA) with the *midas* command packages to generate forest plots of sensitivity and specificity with 95% CI and carry out meta-analyses and meta-regression analyses.

## 3. Discussion

Abdominal tuberculosis is a severe EPTB, which can lead to very serious consequences. Early diagnosis and treatment are very important for the prognosis and the diagnosis of abdominal tuberculosis is still difficult. NAATs play an important role in the diagnosis of TB. It can improve the rate of early diagnosis of tuberculosis. To the best of our knowledge, this will be the first diagnostic meta-analysis for the diagnostic efficacy of NAATs for abdominal tuberculosis. We hope that the results of the study will help clinicians and patients to further understand the role of NAATs in the diagnosis of abdominal tuberculosis. The strength of the body of evidence will be assessed using The Grading of Recommendations Assessment, Development and Evaluation (GRADE) guideline.

## Supporting information

S1 Checklist Preferred Reporting Items for Systematic review and Meta-Analysis Protocols (PRISMA-P checklist). (DOC)
